# The Principles and Practice of Obstetrical Medicine and Surgery

**Published:** 1856-01

**Authors:** 


*The Principles and Practice of Obstetrical Medicine and Surgery, in reference to the process of Parturition.* By Francis H. Ramsbatham, M. D., &c., &c. Edited by Wm. V. Keating, M. D., A. M. &c., p. 648. Phila—Blanchard & Lea, 1855.

Too much cannot be said in praise of this beautiful work. Produced by a distinguished practitioner and teacher, it bears the stamp of the highest practical tact, the soundest philosophy, and truest science. Well arranged, full and thorough, it commends itself to the student, the practitioner, and teacher. A familiarity with its contents may be justly plead as evidence of proficiency in all it professes to teach. The illustrations are well designed and beautifully executed. The book, in every way, is not to be easily equalled. The well known publishers have outdone themselves, which is hard to do, in their department. We most heartily commend it to the profession generally.

